# Preclinical Assessment of Living Therapeutic Materials: State-of-Art and Challenges

**DOI:** 10.1021/acsbiomaterials.5c00247

**Published:** 2025-04-15

**Authors:** Krupansh Desai, Joëlle Mekontso, Ketaki Deshpande, Sara Trujillo

**Affiliations:** † INM-Leibniz Institute for New Materials, 66123 Saarbrucken, Germany; ‡ Chemistry Department, Saarland University, 66123 Saarbrucken, Germany

**Keywords:** Living Therapeutic Materials, preclinical assessment, drug delivery, biocompatibility

## Abstract

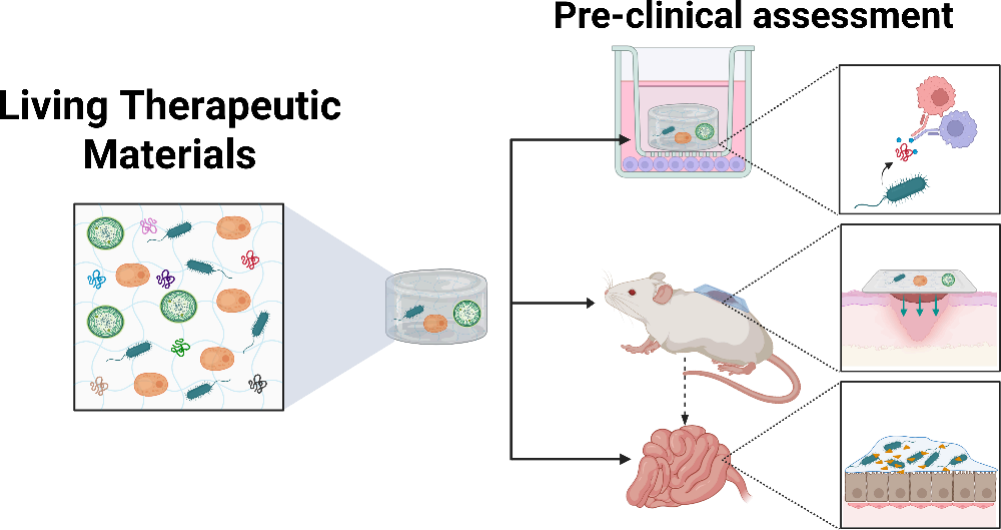

Living Therapeutic Materials represent a promising technology to tackle therapeutic problems that classical materials cannot address. Despite the advancements on new functions of these devices, new applications, and new fabrication methods, the preclinical evaluation of Living Therapeutic Materials is still very limited and new challenges appear when incorporating the living devices in contact with the host. This is a critical bottleneck in the path to translation to the clinic. Therefore, we have compiled the literature on Living Therapeutic Materials, with a focus on microorganism-based living therapeutic materials, and summarized the investigations carried out to assess their biocompatibility, safety, and efficacy. We have split the investigations in three parts: *in vitro*, *ex vivo*, and *in vivo* assessments, where we describe common practices and remaining challenges.

## Introduction

**Figure 1:**
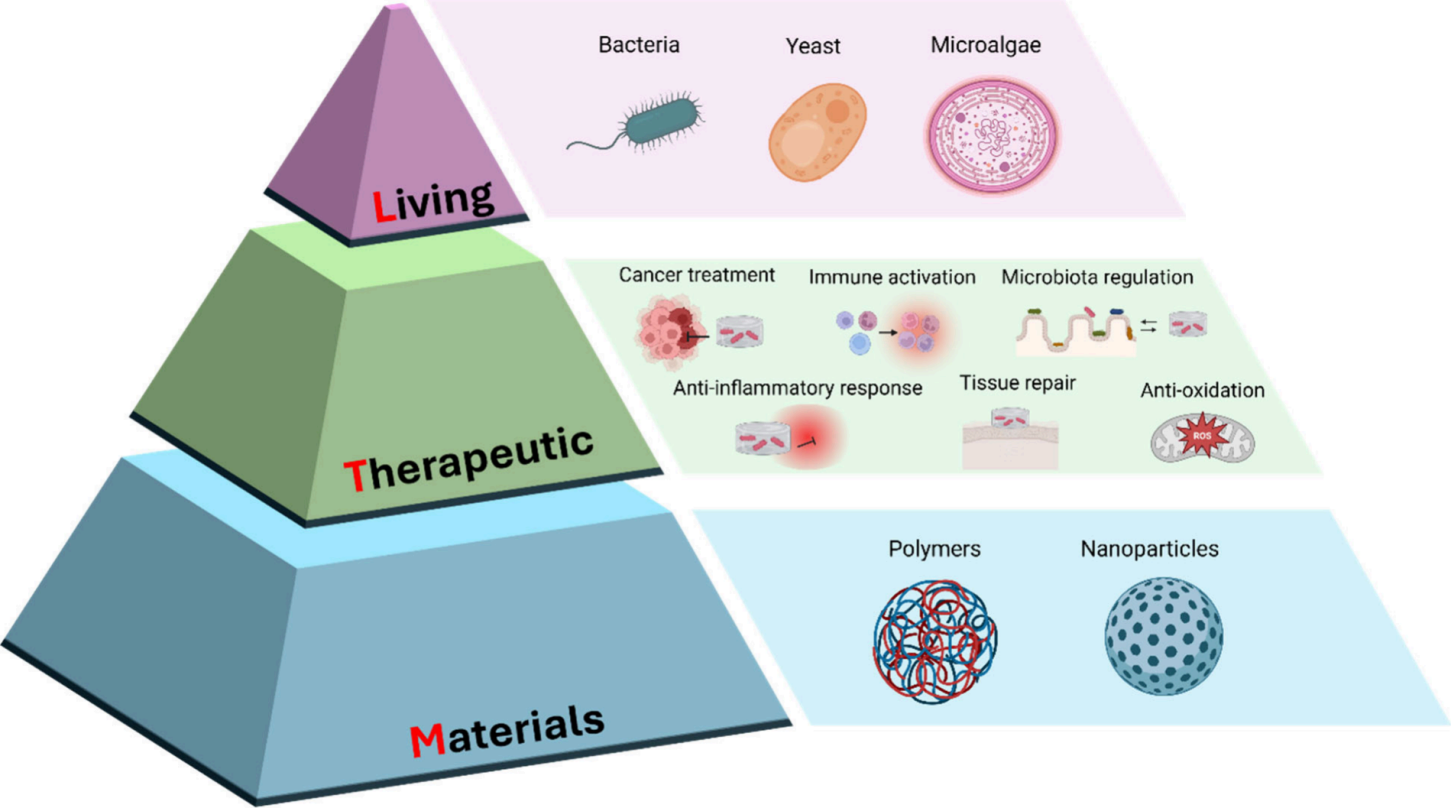
Schematic of Living Therapeutic Materials, which comprise living components, such as bacteria, yeast, etc., and materials, such as polymers or nanoparticles, for a variety of therapeutic applications.

Advances in the past decades in materials science, biofabrication methods, and synthetic biology have given rise to new fields like living materials. A living material is a class of biohybrid composite with living elements, including bacteria, yeasts, fungi, and mammalian cells, integrated with nonliving components.^1−6^ These materials combine the advantages of both living and nonliving components to generate novel functions such as responses to environmental parameters and syntheses of complex biomolecules.^7^ The nonliving aspect combines diverse chemistries and manufacturing techniques to support or enhance the functions of the living part.^6^ Living materials as therapeutics (Living Therapeutic Materials, LTMs) bring revolutionary options to diagnostic and therapeutic practice, offering a solution to life-concerning issues by life itself (Figure 1). Living Therapeutic Materials are revolutionizing classical drug delivery devices, as they can produce therapeutics long-term, *in situ*, and on demand. This represents a more sustainable way for treatment. To realize Living Therapeutic Materials in the clinic, more preclinical studies need to be carried out so the concerns in terms of safety are well understood and their capacity as a more efficient delivery system is assessed. In the past decade, there has been a rise in the number of proof-of-concept LTMs and yet, the preclinical investigation of these materials is just starting.

In this paper, we present a summary of the state-of-the-art preclinical assessment of LTMs with a focus on applications involving direct contact with the host, such as drug delivery. We will focus specifically on LTMs encompassing microorganisms and/or nonmammalian cells, as classical tissue engineering has covered most of the preclinical evaluation challenges of mammalian cell application in medicine.^8−10^

We have classified the preclinical assessment of LTMs in three categories, *in vitro*, *ex vivo*, and *in vivo* investigations, which we will use to critically review the information available on LTMs interfacing the host. For each category, we identify common practices and unsolved challenges that will need to be addressed in the future.

## Living Therapeutic Materials vs Live Biotherapeutic Products

LTMs have been fabricated using viruses, fungi, bacteria, or yeast trapped in a polymeric matrix such as a hydrogel network. However, they differ from Live Biotherapeutic Products (LBPs). LBPs are defined by the European Directorate for the Quality of Medicines and Healthcare as “medicinal products, excluding vaccines, containing living microorganisms such as bacteria or yeasts, which have a positive influence on the health and physiology of the host”. Some of the most common species are *Lactobacilli*, *Bifidobacteria*, and *Saccharomyces cerevisiae*. LBPs are by nature considered medicinal products, as the active substances are the live microorganisms (this is true even if the microorganism has been engineered). There are several reviews on LBP use and challenges from which LTM developers can learn.^11,12^ One challenge that LTMs can take advantage from resides on coatings or encapsulation methodologies to deliver probiotics intact. This is particularly interesting for therapies directed to the gut, as some probiotics might not survive the harsh conditions of the gastrointestinal tract, such as gastric acid, bile salt, or peristaltic movements.^13^ These are normally incorporated in LTMs through the nonliving components, which are usually polymer-based, such as alginate, dextran, or Pluronic.^14,15^ Critical features include biosafety (i.e., limiting the living component from interfacing directly with the host and protecting both the microorganism of the LTM and the host from unwanted interactions). For example, Tang et al. designed a system that incorporates a biocompatible multilayer shell and an alginate-based core for the encapsulation of genetically modified microorganisms. With this system, the encapsulated microorganisms were unable to escape the matrix and were protected from potential harmful parameters such as low pH and antibiotics.^16^ Regarding their role in drug release, hydrogels enable a programmable and controlled release of therapeutics. Wang et al. designed a hydrogel that forms *in situ* after injection at the site of a tumor and releases gemcitabine and an anti-PD-L1 blocking antibody in a programmable manner with the purpose of degrading reactive oxygen species.^17^ Polymer-based matrices have also been used to improve the bioavailability of some drugs, as well as the therapeutic outcome in the treatment of diseases such as infections and cancer.^18−20^ Further, parameters such as the charge or hydrophilicity of hydrogels could be tuned to enhance cell adhesion.^14,21^

Contrary to LBPs, in Living Therapeutic Materials, the active substance might not be the microorganism but a molecule or molecules that are produced by the microorganism (engineered or not). This small but important difference might make LTMs a combination product, in particular, combined advanced therapy medicinal products (ATMPs).^22^ For instance, an LTM comprising an endotoxin-free variant of *Escherichia coli* (*ClearColi*) is encapsulated in agarose hydrogels. The bacteria are genetically modified to produce the antimicrobial and antitumoral drug deoxyviolacein in a light-regulated manner.^23^ Deoxyviolacein is produced by *Chromobacterium violaceum*, and its mechanism of action is thought to be through the accumulation of the compound on the bacterial cell membrane, which initiates its disruption, eventually leading to cell lysis. The osmotic balance of the cells is disrupted, inducing an inhibition of cell growth or cell death.^24^ Therefore, an LTM producing deoxyviolacein will have its effects through the mechanism of action of deoxyviolacein, the active compound, and this would be independent of the microorganism used to produce it, unless there is a synergistic effect found between the LTM and the therapeutic produced.

As combination ATMPs, Living Therapeutic Materials need to address their specific risks according to their intended use, administration mode, and safety, among others. The efficacy of the treatment of an LTM will be defined by the mode of action (MoA) of the therapeutic released and the ability of the LTM to hit the therapeutic window with an adequate dose, but not by the microorganism itself, as it is envisioned as a drug depot within the product. Concerns regarding quality, upscaling, Good Manufacturing Practices (GMP), batch-to-batch variations, or storage will also need to be considered during the development of LTMs.

The right classification of any new therapeutic product is an important step in their path to translation, as LTMs and LBPs are complex products, and there are borderline scenarios that can make them difficult to classify.^22^ For example, the classification of a genetically modified bacteria that expresses a human gene sequence in the patient after its administration could be classified as a gene therapy medicinal product at first. However, if the repair, replacement, addition, or deletion of the genetic sequence is not done at the level of the human cell, then it should not be classified as a gene therapy medicinal product. Another borderline scenario relates to products that are modified by adding a mRNA sequence such as an immune cell electroporated with mRNA *in vitro* and administered to elicit a specific immune response. Due to the relatively short half-life of mRNA and the fact that there might be no residual mRNA at the time of administration, it can be argued that the recombinant nucleic acid is not actually administered. Another borderline scenario is between combined or noncombined ATMPs. If the medical device, such as the use of a matrix, is an integral part of the final product (combined) or if the combined component is not used as a medical device in the final formulation (not combined). For example, human endothelial cells were cultured in a gelatin matrix and used to treat vascular injuries. The underlying MoA is based on the activity of endothelial cells releasing biological molecules, but the gel matrix, which is a medical device, is seeded with the cells and contributes to the formulation of the final product. The gel matrix is remodeled by the cells, so the manufacturing process uses the matrix in a different way than its intended use when considered as a medical device. In this formulation, the matrix would not be considered as a medical device, therefore, the product is not a combined ATMP.

### Path to Translation

**Figure 2:**
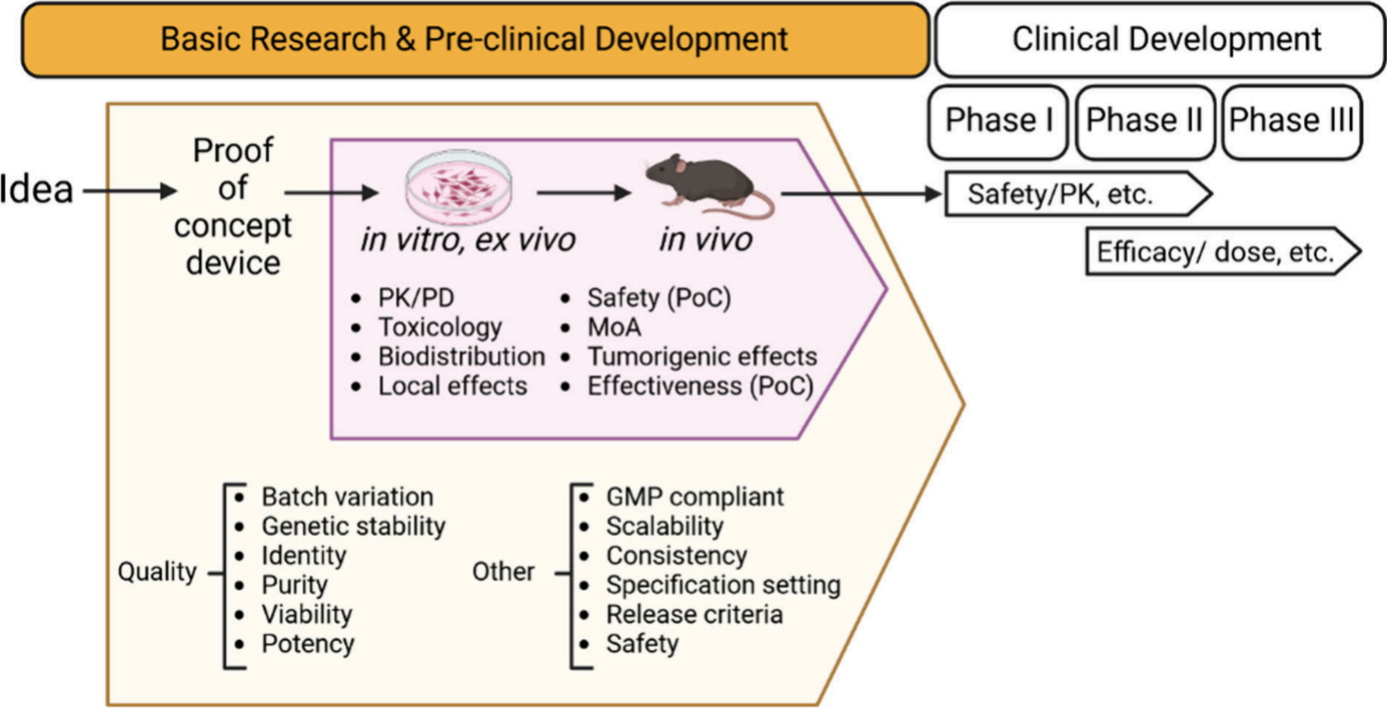
Path to translation for Living Therapeutic Materials. From the idea of the LTM to the proof of concept (PoC) device that might be tested in clinical assays, there are many parameters that need to be characterized during the preclinical development of the product, from the quality of the device (identity, purity, viability, potency, etc.) to toxicological studies and mechanism of action (MoA).

Prior to starting clinical trials, several factors need to be addressed and characterized.^25^ A newly developed ATMP must be proven safe and effective in a planned preclinical assessment program (Figure 2). This includes studies that address pharmacokinetics/pharmacodynamics (PK/PD) and toxicology.^26^ For an ATMP to be proven safe, the risk-benefit profile must be assessed as acceptable for the target application (e.g., disease), and it must be characterized to reduce uncertainty from batch-to-batch variations while ensuring the expected function. Genetic stability must also be confirmed. Defining the identity, viability, purity, and potency of the ATMP is important to ensure consistency between batches and to avoid possible differences in effectiveness.^27^

Experimentation using animal models should be utilized to evaluate the biodistribution of the ATMP to principal organs (i.e., brain, liver, spleen, kidneys, lungs, gonads). These studies are also aimed at identifying target organs and assessing local effects.^28^ Confirmation of the expected MoA of the therapeutic needs to be made. Animal experiments should also prove the safety profile of ATMP and lack of tumorigenic effects. Effective treatment might be proven with animal experiments, but in many cases, there are no animal models for the specific condition to be treated by the ATMP. In these cases, preclinical studies might be performed in different animal models (e.g., that present different symptoms, that mimic better the human disease scenario, etc.).

Once there is a proof-of-concept ATMP, this can be developed into a product for clinical use.^29^ The product must comply with legal requirements for human use, such as manufacturing in compliance with GMP standards in GMP-accredited facilities. Quality attributes of all raw materials and excipients must be suitable for clinical use. Quality documentation should include batch records and expiration dates. Sometimes ATMPs require ingredients such as culture reagents that must be replaced by a defined, GMP-compliant substitute. Upscaling to yield cell numbers for clinical use might be required and processes should be defined. Upscaling might affect the cost and quality of the final product, and therefore, the identity, potency, purity, and safety of the cells used for the ATMP must be maintained after scaling-up. Reproducibility, safety, and consistency of the product should be met to the specifications of the final product. Cell isolation and expansion, cell storage, lot sizes, and other methodologies used during the manufacturing process should be established in a way that minimizes variability. Release criteria must be determined and met in terms of undesired microbiological growth, physicochemical properties, dose range, cell viability, endotoxin levels, or others. Stability periods must also be determined based on cell stability and/or product life cycle.

## *In Vitro* Investigations in Living Therapeutic Materials

*In vitro* assays are highly valuable in drug discovery and pharmacological research evaluating a large number of candidates in different conditions. *In vitro* testing enhances the knowledge of the toxicity and biocompatibility of LTMs by mimicking biological responses in mammalian cell cultures and enabling the assessment of LTM in defined circumstances.^30^ The data obtained from *in vitro* experiments are relatively inexpensive and technically affordable. It is noteworthy that most of the *in vitro* investigations of LTMs are based on 2D cell culture models.

### Cell Viability

**Figure 3:**
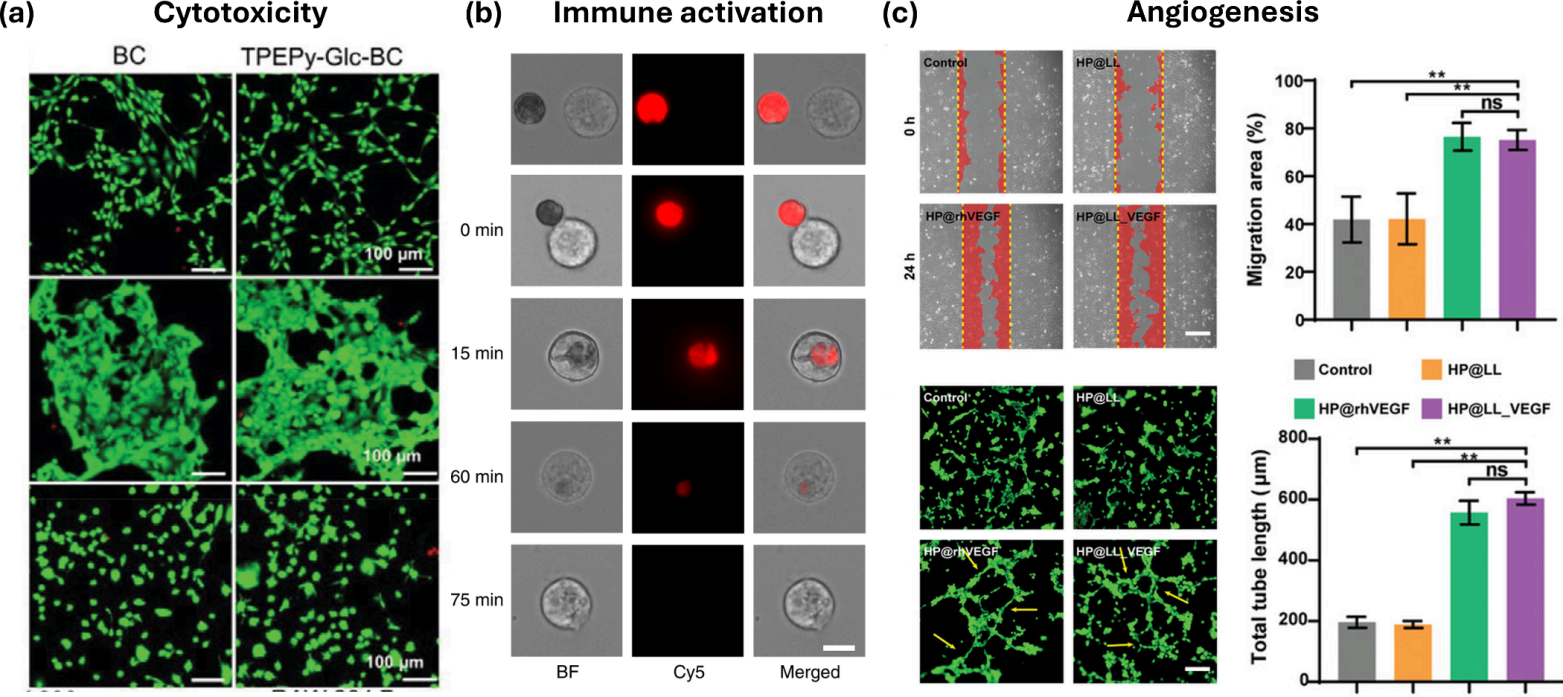
In vitro biocompatibility assessment of LTMs. (a) Fluorescence images of FDA/PI-stained NIH-3T3 cells (first row), HEK 293 cells (second row), and RAW 264.7 cells (third row) treated with bacterial cellulose (BC) and photosensitizer-grafted BC (TPEPy-Glc-BC) under light irradiation. Scale bar: 100 μm.^33^ (b) Representative images of a macrophage phagocytosis assay, where images show red algae-nanoparticle robots incubated with macrophages at various stages of their interaction. Scale bar: 10 μm.^35^ (c) Representative images and quantification of HUVECs migration where red area indicates migrated cells (scale bar: 50 μm). (d) Representative images and quantitative analysis of tube formation assay in HUVECs stained with calcein-AM (green) where yellow arrows indicate the structure of vessel tubes (scale bar: 50 μm).^43^ Reproduced with permission.

The most fundamental yet critical assays performed on any material intended to come in contact with humans are biocompatibility and cytotoxicity assays. The biocompatibility assay refers to the study of a biological effect caused by direct or indirect interactions of a specific material with a mammalian cell line or tissue;^31^ on the other hand, cytotoxicity assays refer to the evaluation of cell damage caused by a foreign substance or material.^32^ These assays are widely used in drug discovery to screen for ideal candidates and allow for high-throughput investigations. The most widely used cytotoxicity assays performed on LTMs are 3-(4,5-dimethylthazolk-2-yl)-2,5-diphenyl tetrazolium bromide (MTT) assay, live/dead staining, and cell counting kit (CCK-8) assay, usually at time points of 12 to 24 h, as summarized in Table 1. For example, Liu et al. investigated *Komagataeibacter sucrofermentans*-based bacterial living material at 24, 48, and 72 h with fibroblasts, embryonic cells, and macrophages via MTT assay, SEM images, and live/dead staining (Figure 3a) show the suitability of this LTM to be in contact with different cells lines.^33^

**Table 1:** *In Vitro* Assays Were Performed on LTMs

assay type	cells used	time (h)	LTM	therapeutic application	ref
MTT assay	4T1s, CT26s, 3T3s	24	*T. denitrificans*, hyaluronic acid	drug delivery	(47)
live/dead staining	L929s	24			
MTT assay	4T1s, SKOV 3s, HEK293s, HepG2s, fibroblasts, Jurkat Ts, J774s	24	*C. vulgaris*, red blood cell membranes	delivery of oxygen	(48)
MTT assay	rat small intestinal epithelium cells	24	*S. platensis*, amifostine	drug delivery	(40)
live/dead, ROS production, DNA double-strand breaks, IF	IEC-6s, CT26s	1			
CCK8 assay	4T1s	24	*S. oneidensis* MR-1, MOF, DOX, HA	drug delivery	(36)
ROS detection		48			
MTT assay	CT26s	12	*S. cerevisiae*, PLGA NPs, Temozolomide, o6-benzylguanine chitosan	drug delivery	(49)
DNA repair protein expression, Western blot		48			
MTT assay	CT26s, HT29s, HCT116s	48	phages, dextran NPs, irinotecan	drug delivery	(50)
MTT assay, live/dead	HPFs, A549s, A549Ts	24	*E. coli*, oligo(phenylene-vinylene)-alkyne	drug carrier	(51)
copper conversion	HPFs, A549s, A549Ts	12			
GSH content	HPFs, A549Ts	12			
oxygenation and SOSG detection, CCK8 assay	4T1s	12	*S. elongatus* PCC 7942, Ce6, protoporphyrin	delivery of oxygen	(52)
live/dead staining	4T1s	24	*S. elongatus* UTEX 2973, mesoporous silica NPs ICG, alginate	delivery of oxygen	(38)
ROS detection		24			
Annexin V and PI staining		24			
CCK8 assay		24			
oxygenation and SOSG detection, CCK8 assay	4T1s	12	*S. elongatus* PCC 7942, black phosphorus nanosheets	delivery of oxygen	(53)
Annexin V and PI staining		12			
MTT assay, live/dead staining, Annexin V and PI staining	CT26s		*E. coli*, AuNPs	drug delivery	(39)
oxidative stress	4T1s	8	*L. acidophilus*, lactate oxidase, tirapazamine, liposomes		
CCK8 assay	4T1s	24			
live/dead staining	4T1s	12			
flow cytometry, cytokine detection	murine bone-marrow-derived dendritic cells	24			
MTT assay	CT26s	24	*S. oneidensis* MR-1, MnO_2_ nanoflowers	tumor microenvironment regulation	(54)
MTT assay	CT26s, RAW264.7s	24	*S. cerevisiae*, ZIF, alcohol dehydrogenase	temulence therapy	(55)
immune activation, cytokine detection, iNOS detection	RAW264.7s	12			
chemotaxis	RAW264.7s	2			
MTT assay	CT26s	24	*S. cerevisiae*, MOF, LOX	tumor treatment	(56)
live/dead staining		24			
glucose concentration		24			
lactate concentration		24			
H_2_O_2_ detection		24			
autophagy staining		24			
ATP content		24			
CCK8 assay	Caco-2s	24	EcN, TA, Fe(III)	drug delivery	(57)
CCK8 assay	HCT116s	24	EcN, HA-poly(propylene sulfide) NPs	ROS scavenging	(58)
proliferation, migration, and tube formation assay	HUVECs	24	*L. lactis*, heparin-poloxamer	drug delivery	(43)
polarization	BMDMs	24			
tube formation assay	HUVECs	16	*E. coli*, Pluronic-diacrylate	drug delivery	(42)
CCK8 live dead	HEK293Ts	24	*S. elongatus*, alginate, chitosa, PEGDA	drug delivery	(59)
cytokine detection	RAW246.7s	12	*Chlorella*, *W. cibaria*, alginate	gas therapy	(60)
live/dead staining	HSFs	12			
tube formation assay	HUVECs	12, 24			
scratch wound healing assay	HaCaTs	24			
CellTiter proliferation assay	J774A.1s	24	*C. reinhardtii*, neutrophil membrane, PLGA NPs	drug delivery	(35)
macrophage phagocytosis	J774A.1s	24, 48, 72			
cytokine detection	PBMCs	24, 48, 72	*ClearColi*, Pluronic-diacrylate		(61)
CCK8	VEROs	48	*E. coli*, chitosan	drug delivery	(62)
live/dead staining	Caco-2	48	EcN, silica-coated NdFeB microparticles, PVA	living sensor	(63)

### Immune Activation

The interaction of LTMs with the immune system was evaluated by immunomodulatory assays *in vitro*. The most popular assay is the macrophage polarization assay. Macrophage polarization is a process in which macrophages can polarize into either pro-inflammatory (M1) or anti-inflammatory (M2) phenotypes.^34^ For instance, Zhang et al. used murine macrophages RAW264.7 to investigate whether their LTM could activate macrophages or not. To do that, they used a transwell system, having the macrophages in the bottom, outside the transwell, and the LTM in the transwell. Flow cytometry was used to quantify the presence of key markers to characterize the macrophage phenotype, and immunofluorescence of iNOS and arginase-1 together with ELISA of key cytokines was also performed. Chemotaxis assay to assess potential LTM phagocytosis by macrophages (Figure 3b) was also performed.^35^ Reactive oxygen species quantification is also a key inflammatory marker that might be involved in the MoA of certain LTMs and has been measured mostly in carcinoma cell lines.^36−39^

### Invasion and Translocation

*In vitro* assays have also been implemented to assess whether LTMs can reduce inflammation in the gut. For example, Praveschotinunt et al. engineered *Escherichia coli* Nissle 1917 (EcN) to produce a self-assembled curli fiber matrix fused to trefoil factors (TFFs) *in situ* within the gastrointestinal tract. TFFs are known to promote intestinal barrier function and epithelial restitution. Invasion, translocation, and barrier function assays were conducted on Caco-2 cells, which failed to show an increased invasiveness into polarized Caco-2 monolayers for both unmodified EcN and EcN-derived strains, and there was barely any translocation for all the EcN-derived strains. Hence, it was concluded that bacteria remained nonpathogenic despite the genetical engineering.^40^ For the invasion assays, Caco-2 cells were cocultured with LTMs for 2 h. After that, bacteria were aspirated and Caco-2 cells were lysed. Lysates were plated to count the colony-forming units (CFUs) of bacteria that invaded the cells. For the translocation assays, Caco-2 cells in transwells were cocultured apically with the LTM for 5 h. The medium was plated, and CFUs that translocated were counted. Epithelial integrity of polarized Caco-2 cells was also determined by TEER values and cytokine secretion via ELISA.

### Angiogenesis

Angiogenesis is a complex process necessary during wound healing.^41^ To study this process, endothelial cells *in vitro* were used together with LTMs. For instance, Dhakane et al. evaluated an optoresponsive LTM secreting a vascular endothelial growth factor mimetic peptide. Human Umbilical Vein Endothelial Cells (HUVECs) on 2D Matrigel was selected as a model to measure angiogenic potential.^42^ Likewise, Lu et al. used HUVECs as a model to evaluate LTM potential to enhance proliferation, migration, and tube formation in 2D.^43^ Chen et al. used HaCaT cells (human keratinocytes) in a scratch wound healing *in vitro* assay and HUVECs to assess angiogenesis of their LTM (Figure 3c,d). The time points used on these assays are relatively early, around the 24 h mark, which goes hand in hand with the rapid remodeling that HUVECs do when applied on 2D surfaces *in vitro*.

### Common Practices and Challenges

Most *in vitro* assays performed in LTMs are based on toxicity assessment of LTMs at early time points with different cell lines and in 2D. Mostly the MTT assay is performed as it is one of the recommended assays in international guidelines for biocompatibility assessment of medical devices (ISO 10993-4). Other viability measurements have been applied to LTMs such as live/dead staining, apoptosis quantification, or CCK-8 assay for proliferation. Other general assays involving the effect of LTMs when in contact with the immune system are scarce in the LTM literature. Some examples involve the use of murine macrophages at early time points to characterize macrophage phenotype (M1/M2). Caco-2 cells are also widely used for *in vitro* investigation of LTMs applied to the gut. Cytotoxicity, invasion, and translocation are the most used assays. To investigate the angiogenic potential of LTMs, HUVECs are primarily used in classical migration, tube formation assays.

The coculture of LTMs with mammalian cells for the investigation of host responses has not been highly exploited. There are a battery of well-developed assays used in other fields, such as tissue repair and drug discovery, that could be easily implemented. The time points used in literature are relatively early time points (up to 72 h), which raises the question if LTMs can be cocultured *in vitro* for longer. This could be a huge challenge considering that microbe-based LTMs might grow faster and consume more nutrients compared to mammalian cells, making the maintenance of the coculture more difficult at longer time points. Some strategies are being applied to achieve longer coculture time points. For example, Petaroudi et al. and Hay et al. cultured a biofilm of *L. lactis* with mesenchymal stem cells for 14 and 28 days, respectively. To achieve this, they employed a cocktail of bacteriostatic and antibiotics that slowed down the metabolic activity of the bacteria (i.e., tetracycline, sulfamethoxazole, and erythromycin).^44,45^ Another critical challenge is the selection of a common culture medium that can support both LTM and the host in vitro model, which could be utilized to slow down the growth of the living component of the LTM, e.g., by selecting a nonoptimal culture medium. In many cases, media selection for cocultures is not specified or, if it is, the quantification of performance and functionality of the LTM is not investigated on these media. Other strategies to slow growth could come from engineering physical entrapment in the matrices. For example, Bhusari et al. showed the differences in *ClearColi* growth with different viscoelastic properties of the material.^46^

## *Ex Vivo* Assays in Living Therapeutic Materials

*Ex vivo* experiments are conducted on cells, tissues or organs, which have been isolated from their natural biological state.^64^ These models aim to mimic some aspects of the structural complexity and physiology of the living organism as closely as possible.^65^ Some aspects of the preclinical assessment of biomaterials can be performed using *ex vivo* models, which represent a promising alternative to animal studies during the development of the therapeutic since they enable the study of higher numbers of tests using standardized conditions.^66^ Some techniques have been reported focusing on different aspects of biocompatibility, depending on the intended applications.^67−69^ Examples using *ex vivo* assays for the assessment of LTMs are summarized in Table 2 and discussed, depending on application.

**Table 2:** *Ex Vivo* Assays Used in LTMs

animal	tissue/organ	assay	LTM	therapeutic application	ref
mouse	intestine	adhesion	*L. reuteri*, chitosan	drug delivery	(70)
mouse	blood	hemolysis	*T. denitrificans*, hyaluronic acid	drug delivery	(47)
mouse	tumor	ROS production	*C. vulgaris*, red blood cell membranes	delivery of oxygen	(48)
mouse	blood	hemolysis			
pig	stomach, intestine, colon, cecum	adhesion	EcN, TA, Fe(III)	drug delivery	(57)
mouse	colon	adhesion	EcN, mucin, TA	ROS scavenging	(71)
mouse	lung	retention	*C. reinhardii*, neutrophil membrane, PLGA NPs	drug delivery	(57)
horse	blood	hemolysis	EcN, silica-coated NdFeB microparticles, PVA	living sensor	(63)

### Mucoadhesion

Living Therapeutic Materials have been engineered to treat the gut.^6,71−73^ For these applications, *ex vivo* assays have been relevant to understand and quantify how the LTM adheres to the surface of the specific parts of the gastrointestinal tract (GI) or the viability of the LTM passing through the GI tract. For example, Luo et al. used the *ex vivo* porcine GI tract to evaluate the suitability of the coating added to the encoding bacteria to adhere to the mucosal layer. They also evaluated adhesion to nasal mucosa and skin using the same animal model. The engineered coating improved bacterial adhesion by 45-fold compared to uncoated control.^57^ Similarly, Kaur et al. investigated the adhesion of their LTM to murine intestine using *ex vivo* culture. They monitored the anchorage of the LTM using confocal microscopy and scanning electron microscopy.^70^ The same strategy was used by Yang et al. to measure adhesion of their LTM to murine stomach, small intestine, colon, and cecum, in parallel.^71^

### Hemocompatibility

*Ex vivo* assays are widely used to study hemocompatibility and blood clotting. For this, hemolytic assays are used following international standards (ISO 10993-4). Here, whole blood is collected and centrifuged to gather the erythrocyte-free plasma portion. Hemoglobin concentration is then measured spectrophotometrically. Then, blood is placed in direct contact with the LTMs (37 °C, 3 h, and shaking) and the hemoglobin released into the solution is measured. Controls are used following guidelines (ASTM F-756), with positive control being plastisol and negative control being high-density polyethylene, and negative controls were PBS^47^ and deionized water.^48^ Qiao et al. performed hemolysis tests on their engineered algae system that delivered oxygen to tumors, showing that their LTM was hemocompatible.^47^ Similarly, Li et al. showed that their thiobacillus dentrificans-hyaluronic acid-based LTM was hemocompatible.^48^ It is noteworthy that the positive controls used, despite generating hemolysis levels over 90%, have not been validated as stated in the guidelines.

### Common Practices and Challenges

In general, the use of *ex vivo* assays is not common, and researchers typically move from cellular *in vitro* models to *in vivo* models. This could be due to difficulties in explanting techniques, lack of access to surplus material, or problems establishing assays. Common practice is the use of an explanted GI tract to study LTM adhesion and investigate the mucosal layer or other explanted organs/tissues (i.e., lung, tumor) to assess LTM effects over time. Some established *ex vivo* assays, such as the Simulator of the Human Intestinal Microbial Ecosystem (SHIME), could help LTM developers to study human microbiota *in vitro*. The study of the microbiome is essential to understanding how the LTM might interact with or change the natural flora. However, the gut microbiota cannot be mimicked accurately in any animal model available.^74^ In these cases, the use of the *ex vivo* gastrointestinal SHIME model could be helpful.^75^ LTM developers have not yet used this kind of approach to study the gut microbiome, but there are examples using LBPs. For example, *Christensenella minuta* was investigated as LBP for obesity treatment in preclinical models.^76^ There, Mazier and colleagues investigated the effects of *C. minuta* on the gut microbiota using the SHIME model inoculated with obese faecal samples. The SHIME model showed that the antiobesity effects observed were associated with variations of the Firmicutes/Bacteroidetes ratio in the intestinal region.

For other applications, such as the study of potential pro- or antiangiogenic responses, the chick chorioallantoic membrane (CAM) assay could be useful.^77−79^ The CAM assay makes use of the extraembryonic chorioallantoic membrane of the chick embryo, which is well vascularized. The *ex ovo* CAM assay has been used for the biocompatibility assessment of biosensors. For example, Valdes et al. explored the feasibility of the *ex ovo* CAM model in testing the functionality of an acetaminophen sensor. This sensor was incorporated into the CAM of an embryo for 7 days. The blood levels of acetaminophen determined with the biosensor reflected the amount of acetaminophen cleared by the chicken embryo.^80^ Klueh et al. also used this model to test the functionality of a biosensor meant for the measurement of glucose concentrations based on the electrochemical oxidation of H_2_O_2_.^81^ These assays can be easily adapted to investigate living biosensors.

*Ex vivo* human skin tissue could be more appropriate than the CAM for certain applications.^82^ Whole skin biopsies allowed for the investigation of individual components in an environment that mimics more closely normal skin. A full-thickness skin culture is an established tool that has been used to understand human skin pathophysiology and the wound healing process. However, there is still a lack of standardization and conformity using these models (i.e., use of different wound types, culture conditions, or support media). *Ex vivo* skin tissue models were used by Luo et al. to investigate LTM adhesion.^57^ In the case of LBPs, the probiotic strain *Lactobacillus reuteri* DSM 17938 was investigated for topical application, showing a reduction in inflammation in a UVB radiation-induced inflammation model.^83^ Here, full-thickness skin from plastic surgery surplus was taken and incubated in Dispase with the epidermis facing down to be peeled off of the explant. Then, different concentrations of *L. reuteri* in RPMI medium with 10% FBS were used as pretreatment to the epidermis. The explant was exposed to UVB radiation, and *L. reuteri* treatment was repeated. Then, several cytokines were quantified, and the gene expression of several markers was measured. Dou et al. used both porcine and human *ex vivo* skin models to investigate a yeast-based curcumin carrier for the delivery of curcumin to the skin for the treatment of inflammatory skin diseases such as psoriasis.^84^ In this case, skin samples were incubated in transwells with the yeast-based carriers placed on top and using Iscove’s Modified Dulbecco’s Medium with 10% FBS in the bottom well. Biopsies were kept at 32 °C with 5% CO_2_ in the dark. Then, parameters such as yeast binding to the skin surface or curcumin penetration in the skin were quantified.

## *In Vivo* Assays in Living Therapeutic Materials

Preclinical assessment of any medical device must be characterized using animal models. However, regulatory agencies are accepting more data from models that replace animal experimentation, also known as nonanimal models (NAMs).^85^*In vivo* experimentation must always be conducted following the 3Rs (reduction, replacement, and refinement).

Living Therapeutic Materials have been implanted in different animals (i.e., mice, rats, dogs, and pigs) depending on the intended application and relevance to human biology.^86−89^ A summary of the *in vivo* LTM studies can be found in Table 3. Typically, implanted LTMs are investigated for immune response, inflammatory response, and foreign body reaction. Further, *in vivo* assessments are also performed for biodistribution and retention of encapsulated microbes within the host’s key organs and for horizontal gene transfer between encapsulated microbes and the host.^6^

**Table 3:** *In Vivo* Experiments Carried out in LTMs

animal	model	summary of assays	LTM	therapeutic application	ref
mouse	tumor	tumor regression, immune response	*L. reuteri*, chitosan	drug delivery	(70)
		histopathology, renal/hepatic markers			
mouse	tumor	tumor regression, biodistribution	*T. denitrificans*, hyaluronic acid	drug delivery	(47)
mouse	tumor	tumor regression, biodistribution, histopathology	*C. vulgaris*, red blood cell membranes	delivery of oxygen	(48)
mouse	healthy	biodistribution, PK studies, long-term safety	*S. platensis*, amifostine	drug delivery	(40)
mouse	tumor	safety, tumor regression			
		effect on gut microbiota after radiotherapy			
mouse	tumor	tumor regression, detection of Fe and DOX, systemic toxicity	*S. oneidensis* MR-1, MOF, DOX, HA	drug delivery	(36)
mouse	tumor	tumor regression, biodistribution, histopathology	*S. cerevisiae*, PLGA NPs, temozolomide, o6-benzylguanine chitosan	drug delivery	(114)
mouse	tumor	tumor regression, histopathology	phages, dextran NPs, irinotecan	drug delivery	(113)
mouse	healthy	acute and chronic toxicity, biodistribution	*S. elongatus* PCC 7942, Ce6, protoporphyrin	delivery of oxygen	(112)
mouse	tumor	intratumoral oxygenation, histopathology			
mouse	tumor	histopathology, tumor regression, TUNEL	*S. elongatus* UTEX 2973, mesoporous silica NPs ICG, alginate	delivery of oxygen	(38)
mouse	healthy	histopathology, chronic toxicity	*S. elongatus* PCC 7942, black phosporous nanosheets	delivery of oxygen	(110)
mouse	tumor	biodistribution, systemic toxicity, tumor regression, histopathology	*E. coli*, AuNPs	drug delivery	(109)
mouse	tumor	biodistribution, ROS detection, tumor regression	*L. acidophilus*, lactate oxidase, tirapazamine, liposomes		(39)
mouse	tumor	biodistribution, tumor regression, systemic toxicity	*S. oneidensis* MR-1, MnO_2_ nanoflowers	tumor microenvironment regulation	(108)
mouse	tumor	biodistribution, tumor regression, Acetaldehyde generation, immune activation	*S. cerevisiae*, ZIF, alcohol dehydrogenase	temulence therapy	(55)
mouse	tumor	tumor regression, systemic toxicity, Intratumoral glucose and lactate detection	*S. cerevisiae*, MOF, LOX	tumor treatment	(56)
mouse	healthy	bioavailability, intestinal adhesion, colonization	EcN, TA, Fe(III)	drug delivery	(57)
mouse	DSS-induced colitis	histopathology, colon analysis			
mouse	salmonella infection				
mouse	healthy	resistance and residence time, systemic toxicity	EcN, chitosan, alginate	drug delivery	(72)
mouse	IBD model	therapeutic effect, histopathology			
mouse	healthy	colonization, biodistribution, systemic toxicity	EcN, mucin, TA	ROS scavenging	(71)
mouse	healthy	adhesive effect, biodistribution	EcN, HA-poly(propylene sulfide) NPs	ROS scavenging	(58)
mouse	DSS-induced colitis	efficacy, immune activation, histopathology, ROS levels in colon, microbiome analysis			
mouse	healthy	stability of LTM in inflamed colon, long-term retention, efficacy	*B. longum*, Iron single atom catalyst	ROS scavenging	(92)
mouse	DSS induced colitis	histopathology			
mouse	TNBS induced colitis				
dog	acetic acid induced colitis				
mouse	diabetic wound healing	inflammatory response to LTM, biodistribution, efficacy, histological analysis, transcriptome analysis	*L. lactis*, heparin-poloxamer	drug delivery	(43)
rat	wound healing	histological analysis, cytokine detection	*S. elongatus*, alginate, chitosa, PEGDA	drug delivery	(59)
mouse	diabetic wound healing	wound closure quantification	*Chlorella*, *W. cibaria*, alginate	gas therapy	(60)
mouse	dorsal skin flap	skin graft, histological analysis			
mouse	healthy	safety, survival, clearance	*C. reinhardtii*, neutrophil membrane, PLGA NPs	drug delivery	(35)
mouse	wound healing	wound closure, histological analysis	*S. aplatensis*, chitosan, alginate	drug delivery	(97)
mouse	MPTP-induced Parkinson’s model	retention of LTM in GI tract, GABA content in blood, gut microbiome profiling, brain histology, behavioral tests	*L. plantarum*, methacrylic acid, ethyl acrylate	drug delivery	(131)
mouse	MPTP-induced Parkinson’s model	behavioral tests, electrophysiological recordings, histological analysis	*L. lactis*, chitosan, alginate	drug delivery	(132)
mouse	healthy	retention of LTM in GI tract, blood sensing in intestine, histological analysis	EcN, silica-coated NdFeB microparticles, PVA	living sensor	(63)
mouse	healthy	intestinal retention, bioavailability	*B. subtilis*, biofilm-coating	microbial regulation	
pig	healthy				(90)
mouse	*S. aureus* infection				
mouse	*S. aureus* infection	histopathology, wound healing rate, immunohistochemistry	*K. sucrofermentans*, bacterial cellulose	wound repair	(33)
mouse	*C. albicans* pseudohyphael infection	histopathology	*B. subtilis*, Pluronic-based gels	delivery of antifungal agents	(117)
mouse	MRSA infection	histopathology, immunohistochemical analysis, body weight measurement	photosynthetic bacteria, ECM hydrogel	wound repair	(96)
mouse	*C. albicans* infection	histopathology	*B. subtilis*, poly(ethylene glycol) diacrylate (PEGDA)	delivery of antifungal agents	(118)
mouse	MRSA infected diabetic wound healing	wound closure, in vivo bacteria counting of the wounds, histopathology, mRNA expression, Evaluation of blood vessels, collagen deposition, granulation tissue quantification	cyanobacteria, inner layer: oxidized sodium alginate (OSA) and carboxymethyl chitosan (CMCS), outer layer: agarose and CMCS	delivery of oxygen	(100)
mouse	healthy and gastrointestinal bleeding induced model	histopathology, behavioral tests, body weight measurement	EcN, polyvinyl alcohol (PVA) hydrogel	living sensor	(63)
mouse	*S. aureus* infection	wound closure, histopathology, immune cell infiltration, hair follicle formation, collagen deposition	*L. reuteri*, methacrylate-modified hyaluronic acid	wound repair	(93)
mouse	*S. aureus* infection	wound closure, histopathology, blood biochemistry	*S. platensis*, carboxymethyl chitosan	delivery of oxygen	(94)
rat	*S. aureus*-infected diabetic wound	wound closure, histopathology, immunofluorescence, granulation tissue quantification, blood vessel quantification, HIF-1α and collagen deposition quantification	*Chlorella* (algae) and *B. subtilis*, pluronic-based gels	delivery of oxygen	(103)
mouse	healthy	histopathology, body weight	*E. coli*, self-produced matrix	extended residence time in the gut	(91)
mouse	healthy	histopathology, cytokine quantification in serum	*B. subtilis*, self-produced	extended residence time in the gut	(90)
mouse	dextran sodium sulfate (DSS) model of murine colitis	histopathology, ELISA	EcN, self-produced matrix	mucosal healing	(40)
mouse	tumor	histological analysis, serum levels of aspartate aminotransferase (AST), alanine aminotransferase (ALT), blood urea nitrogen (BUN), and creatinine (CRE)	EcN, liposomes	drug delivery	(106)
mouse	tumor	serum levels of aspartate aminotransferase (AST), alanine aminotransferase (ALT), serum creatinine (CRE), and blood urea nitrogen (BUN) in key organs and blood sample	EcN, tannic acid, doxorubicin, Fe(III)	drug delivery	(107)
mouse	healthy	blood analysis and histopathology	*C. Synechocystis* PCC 6803, alginate	bioremediation	(119)
mouse	S. aureus infection	histopathologhy	EcN, BSA hydrogel	wound healing	(95)

### Inflammatory Bowel Diseases

The localization of orally taken therapeutics for prolonging their residence time has been quite challenging due to the short GI transit time. LTM developers have tried to overcome this problem using different strategies. For example, genetically modified *E. coli* Nissle 1917 within magnetic hydrogels that had blood-sensing capabilities were used. Retention time was up to 1 week in the GI tract of mice and no inflammatory response was observed.^63^*Bacillus subtilis* wrapped into self-produced biofilms had enhanced retention time, bioavailability, and mucoadhesion *in vivo* with no elevated immune response.^90^ Studies show that retention and mucoadhesion in the gut could be achieved and immune response was not increased after treatment with the LTMs in either healthy or colitis-induced models.^91,57^ Some LTMs improved inflammation associated with IBD,^72^ and other LTM strategies were used successfully as prophylactics.^58^ When looking at the gut microbiome, some studies found improved diversity and abundance of microbiota.^58^ Cao et al. investigated their bifidobacterium-based LTM for GI treatment.^92^ They studied the efficacy of the LTM in two murine models, a DSS-induced colitis model, which affects the epithelial cells and mucosal layer of the gut, and a 2,4,6-trinitrobenzenesulfonic acid (TNBS)-induced Crohn’s disease (CD) model, showing improved efficacy compared to controls. They also studied the efficacy of LTM in a canine model of ulcerative colitis. This study showed the safety of LTMs in a larger in vivo model. All studies presented similar biocompatibility and low or no immune response, which show that LTM strategies for gut applications are promising. In disease models, LTMs performed well by lowering inflammation. Dosage and administration regimes could be optimized, which could lead to even better treatments. In the future, the possibility of a combination of treatments with different LTMs could also be an option to treat IBD.

### Wound Repair

**Figure 4:**
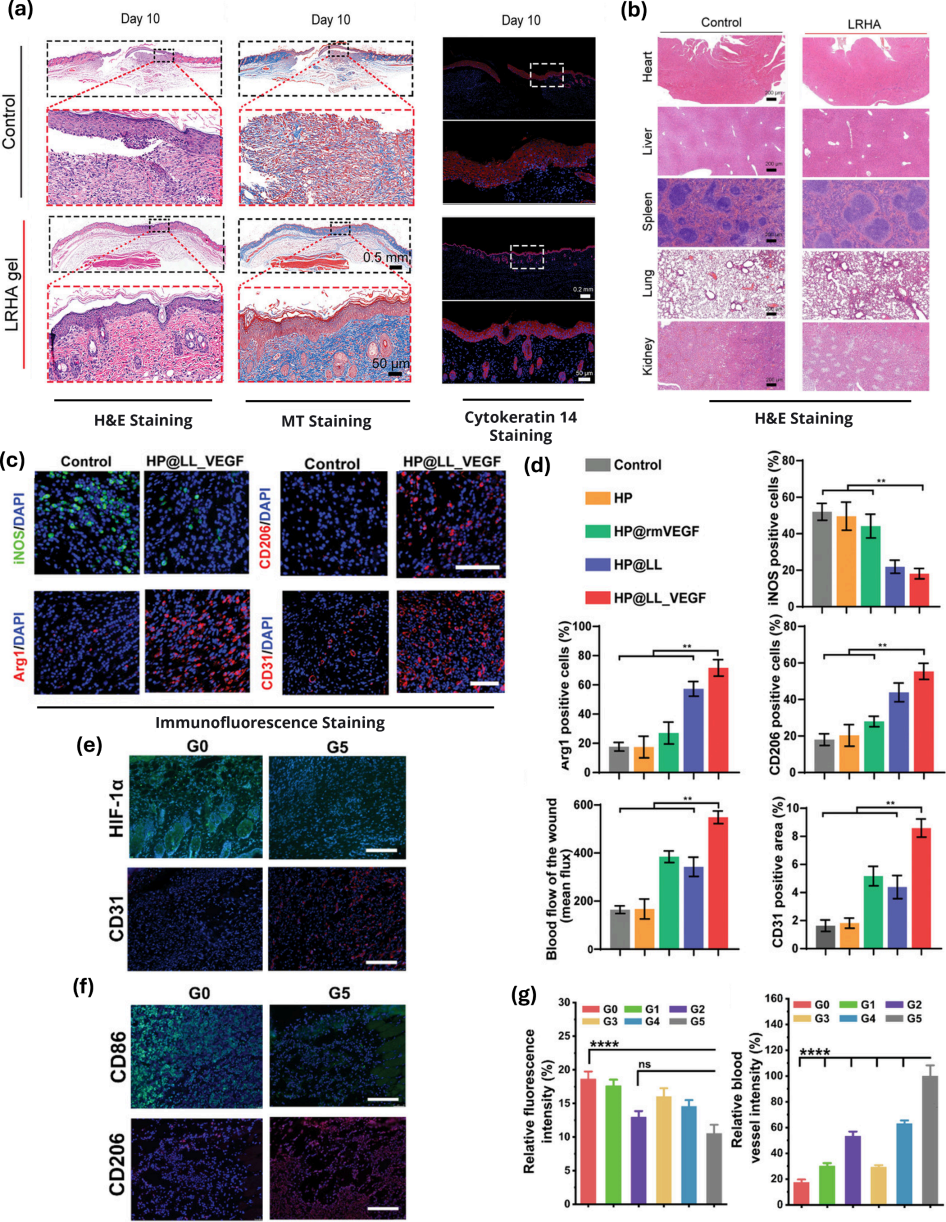
*In vivo* biocompatibility studies on LTMs for wound healing application. (a) Representative images of wound tissue slices stained with Hemotoxylin and eosin (H&E), Masson’s trichrome (MT), and cytokeratin 14 for control (no treatment) and LHRA (*Lactobacillus reuteri* containing HA hydrogels) groups on day 10.^93^ (b) Representative images of H&E staining of key organs after LTM administration.^93^ (c) Representative illustration of iNOS^+^ macrophages (green, M1 marker) and Arg1^+^/CD206^+^ macrophages (red, M2 markers) with cell nuclei (blue) for control (no treatment) and heparin-poloxamer incorporating engineered VEGF-secreting *L. lactis* (HP@LL-VEGF); Scale bar = 100 μm.^43^ (d) Percentage of iNOS^+^, Arg1^+^, and CD206^+^ macrophages (*n* = 3) in different groups; **P < 0.01.^43^ (e) Representative immunofluorescence images of CD31^+^ (red, neovascularization marker) and HIF-1α^+^ (green, hypoxia marker). Scale bars = 50 μm.^100^ (f) Representative immunofluorescence images of CD206^+^ (red, M2 markers) and CD86^+^ (green, M1 marker) for wounds treated without any treatment (G0) and LTMs (G5). Scale bars = 50 μm.^100^ (g) Average HIF-1α expressions and blood vessel densities in wounds treated with different treatments. Data are represented as mean ± standard deviation. Statistical significance at *p < 0.05, **p < 0.01, ***p < 0.001, and ****p < 0.0001.^100^ Reproduced with permission.

Wound repair is a complex but well-orchestrated natural process involving a cascade of cellular and molecular signaling events, such as re-epithelization, inflammation, collagen deposition, granular tissue formation, and vascularization.^41^ LTMs have opened new avenues by incorporating beneficial microorganisms into materials as therapeutics or by genetically engineering various microorganisms to produce bioactive molecules conducive to wound healing. For example, Li et al. reported successful wound healing in rats using living wound dressings that incorporated *Synechococcus elongatus* and *Lactococcus lactis* genetically modified to produce CXCL12. They investigated macrophage polarization and granular tissue formation via cytokine quantification and histological analysis.^59^ Open wounds are prone to infection, which delays the healing, resulting in chronic wounds. Few attempts have been made to tackle this persistent problem for wounds infected with *Staphylococcus aureus* (*S. aureus*)^33,93−95^ or methicillin-resistant *Staphylococcus aureus* (MRSA)^96^ using LTMs. For instance, Ming et al. developed a living bacterial hydrogel for repairing *S. aureus* infected wounds without disturbing the natural skin microbiota in mice. Histopathological analysis showed enhanced collagen deposition and re-epithelialization (Figure 4a,b).^93^ With the same aim, Hu et al. developed an LTM consisting of living microalgae in a gel. They showed that the LTM inhibited MRSA infection and reduced inflammation.^97^

Delivering oxygen is a promising therapy to reduce hypoxic conditions which ameliorate the wound healing process.^98,99^ LTMs have also been employed for this purpose.^94,100^ Li et al. fabricated photosensitizable *Spirulina platensis* containing living hydrogels, producing oxygen naturally and generating reactive oxygen species for destroying *S. aureus* infection upon laser irradiation. Immunohistochemistry analysis of mice tissues for hypoxia-inducible factor-α (HIF-α) and CD31 (neovascularization marker) revealed significantly downregulated and upregulated expressions of these markers compared to the control group, respectively. Additionally, they validated the biosafety of the system by evaluating blood biochemistry and hematology. All the parameters were in the normal range, indicating no negative impact on normal functions of key organs, illustrating outstanding biocompatibility *in vivo*.^94^ Combined gas therapy of nitric oxide and oxygen was the approach developed by Chen and co-workers, where Chlorella and *Weissella* were used in alginate gels to delivery both gases. They used a diabetic wound healing mouse model to prove the efficacy of the LTM and the promotion of angiogenesis in vivo using a flap skin regeneration model in mice.^60^

Systemic problems such as diabetes are also considered one of the main causes of chronic wounds because of strong and persistent inflammatory response hindering blood vessel formation and macrophage polarization in diabetic patients,^101^ among others.^43,94,102,103^ Lu et al. addressed this problem using their LTM immobilizing *Lactococcus lactis*, engineered to produce vascular endothelial growth factor (VEGF) and lactic acid, supporting angiogenesis and macrophage polarization in diabetic mice. Upon staining macrophages for iNOS (M1 marker) and CD206 (M2 marker), they found more M2 marker positive cells compared to M1 marker positive cells for wounds treated with these LTMs compared to the control (Figure 4c,d). Further, histochemical analysis revealed significantly thicker granulation tissue, collagen deposition, and increased CD31 positive area (Figure 4c) compared to control.^43^

Another problem in wound healing of diabetic wounds is that the hyperglycaemic environment and prolonged healing time make the wounds more vulnerable to infection.^104,105^ To treat difficult-to-heal bacteria infected diabetic wounds, Zhu et al. designed an oxygen-producing double layered hydrogel with an outer layer incorporating cyanobacteria for oxygen production and an inner layer with a photosensitizer for wound sterilization upon laser illumination. The H&E staining for *S. aureus* infected wounds in mice revealed successful re-epithelialization at the wound site when treated with LTMs. Fluorescence-labeled expressions were elevated for M1 macrophage markers (Figure 4f) and downregulated for M2 macrophage markers (Figure 4f,e), showing LTMs aided macrophage polarization. Further, the wounds treated with LTMs resulted in notably higher relative blood vessel intensities and lower relative fluorescence intensities compared to the control (Figure 4g), indicating LTMs potential to promote angiogenesis and alleviate hypoxic conditions.^100^

### Cancer Treatment

Bacteria-based LTMs are thought to be a game-changer in cancer therapeutics. These smart systems have been genetically programmed for *in situ* delivery of antitumor drugs, immune response triggering molecules for an immunotherapy, or as a base for phototherapy.^106−108^ For instance, Jiang et al. coated the surface of *E. coli* Nissle 1917 with polymer layers entrapping the doxorubicin drug. Bacteria were genetically modified to trigger bacteria cell lysis in hypoxic environments and releasing antigens stimulating the M1 macrophage transition. The *in vivo* biosafety was evaluated by injecting the bacteria intravenously into mice and monitoring the renal and liver functions. The biomarker levels and histological examinations revealed no adverse effects on major organs.^107^ Most of the LTMs developed for tumor treatment use similar models of mice in which they induce a tumor by injecting tumor cells (e.g., mouse breast cancer 4T1 cells or mouse colon cancer CT26 cells) and they study tumor regression biodistribution and histopathological analysis.^36−39,56,58,109−114^

### Other Applications

The incidents with fungal infection are thought to enormously increase in coming years and drastically impact our ecosystem,^115^ possibly due to global climate change.^116^ Lufton et al. employed their *Bacillus subtilis* incorporated thermoresponsive hydrogels to treat *Candida albicans* infections. Under histological staining, fungus infected mice had no inflammation compared to that of untreated mice, demonstrating antifungal potential and *in vivo* biocompatibility.^117^ Likewise, Wang et al. presented living microneedles (LMNs) entrapping *Bacillus subtilis* for treating fungal infections. For investigating the inflammation potential of these living bacterial systems, a murine model was used. The implanted site for LMNs had very few inflammatory cells in the histological analysis.^118^

LTMs have also emerged as ecofriendly and cost-effective alternatives for bioremediation of heavy metals such as cadmium, copper, and lead from the human body. These metals make their way to humans through food grown in heavy metal contaminated soil resulting from environmental pollution. Sun et al. employed their engineered cyanobacteria-based LTMs to reduce Cd^2+^ levels in mice. Biosafety was confirmed by blood routine examination (e.g., blood urea nitrogen, alanine transaminase, or hematocrit blood test) and pathological analysis of key organs. No significant differences were observed in terms of tissue architecture and blood test results compared to control mice, demonstrating the biocompatibility of the system *in vivo*.^119^

### Common Practices and Challenges

For applications in the gut, common practices include the study of adhesion and retention of LTMs in the GI tract and possible biodistribution. Some authors assess the systemic toxicity and safety of the orally administered LTM, but it is not common practice considering the importance of these experiments toward translation. The most widely used model for gut inflammation is the DSS-induced colitis in mice.^120^ Colitis is one of the major inflammatory bowel diseases (the other being Crohn’s disease), which is characterized by both acute and chronic inflammation of the intestine with multifactorial etiology. DSS is administered in drinking water, and its mechanism of action is unclear. This model is popular due to its rapidity, simplicity, reproducibility, and controllability. An important caveat in DSS-induced colitis is that, unlike human colitis, T and B cells are not required for the development of the disease. The TNBS-induced colitis model is associated with chemically induced damage and T and B cell activation, which might mimic more closely the disease in humans. The use of both models might help in the understanding of the mechanism of action of the LTM treatment. From all the models of colitis disease available, DSS and TNBS-induced colitis are the simplest to generate, as other models involve the generation of knockout mice or the transfer of CD45RB^high^ T cells in lymphopenic mice, which are more technically difficult and expensive to achieve.^121^ The challenges that need to be overcome when using mouse models for studying IBDs are related to the large discrepancy between mice and humans on immune responses, lack of correlation to genetic and environmental diversity in humans, and lack of consideration of dependent variables such as microbiota, smoking, or diet. It is worth mentioning that, for applications in the gut, therapeutics targeting the metabolizing capacity of the natural microbiome are being developed. For example, Li et al. developed nanoparticles containing inulin and oxaliplatin embedded in a hydrogel carrier to be utilized by the microbiota in the gut and stimulate the immune system locally. This strategy proved to be successful for colorectal cancer treatment.^122^

For applications in cancer, the go-to *in vivo* models used by LTM developers are cell-derived xenograft tumor models or CDXs,^123^ which are easy to induce, as they only require the injection of a cancer cell line and some time for it to develop a tumor. Principally, two cell lines have been used to evaluate these modes of LTM treatment: 4T1 cells (mouse breast cancer cells) and CT26 cells (mouse colon cancer cells). Some limitations on these models are that the efficiency of establishment of CDXs varies widely between cancer types and laboratories and that CDX models might take months to establish. These cell-derived models could also be obtained in a more reproducible and high-content way by creating *in vitro* tumoroids using, for example, patient-derived tumor cells. These *in vitro* systems have shown good value in drug screening and could be used for LTMs.^124−126^ Another alternative is the *in ovo* assay using the CAM. The CAM assay is already established in cancer research and is used to assess many aspects of cancer, such as tumor growth, vasculature, invasion, metastatic potential, genomic instability, mutations, or epigenetic reprogramming.^127^ Histologically, CAM-induced tumors have been confirmed to be comparable to the patients’ original tumors, taking 8 days from inoculation to formation of the tumor, which is much faster than any CDX mouse generated.^127^

For applications in wound healing, most LTMs have been studied by using the full-thickness excision wound model in mice. In this model, a wound is created by damaging all the layers of the skin, and it clinically relates to diabetic ulcers. The disadvantages of using a mouse model for wound healing are mainly that the skin is looser and thinner than humans and it has more hair, the healing of the wound occurs via wound contraction, and there are different chemokines and receptors involved compared to human.^128^ The full-thickness excision wound model is widely used in diabetic mouse models, typically db/db, which display obesity, insulin resistance, hyperinsulinemia, and albuminuria. Other genetically modified diabetic mouse models exist, like ob/ob and KK-A^y^.^129^ As an alternative to these models, zebrafish has appeared as an efficient model due to its sequential healing process, where a full-thickness wound can be created very quickly.^130^ The wounds have shown quicker re-epithelialization without depending on coagulation or inflammation and minimal scarring.

## Conclusions and Outlook

In the past decade, we have witnessed the development of a new therapeutic technology based on advances in synthetic biology and material fabrication techniques. Here, we have focused on the state-of-the-art for the preclinical assessment of microorganism-based living therapeutic materials. These materials have the potential to overcome the problems that traditional drug delivery approaches cannot overcome, such as long-term treatment periods. Despite encouraging examples of the applicability of LTMs and innovative strategies for treatments, there are some aspects that the community should pay attention to for translational purposes.

First, the biosafety of LTMs remains a major concern. Although probiotics, yeasts like *S. cerevisiae*, or certain microalgae are edible for humans, the effects that they can cause in weakened populations, e.g., in disease, are not known. On this regard, the Generally Regarded as Safe “GRAS” status is not valid, and safety must be assessed. To do this, dose–response studies should be performed, if possible, in different environmental conditions, diets, and different administration regimes. This will inevitably increase the development cost of the LTM, but it will also improve their acceptability in the medical community. In addition, most LTMs showcase a variety of genetically engineered microbes, of which many contain plasmids. The potential to leak genetic material and transfer genes into the host microbiota should be analyzed to avoid unpredictable outcomes. Some of the microorganisms used in LTMs present virulence factors that can help the microbe colonize the host. Evaluation of the capacity of these microorganisms to colonize a tissue and how the natural microbiota diversity and number changes should be addressed. The development of multiomics technologies will provide more opportunities to tackle this challenge. At present, most studies on LTMs are still focusing on evaluation of possible therapeutic effects using mouse models, which cannot truly reflect the potential application to human beings. For this, more investigations are required in different animal models, but also in human tissue models.

Second, most LTMs lack a clear mechanism of action. Current research has focused on the use of LTMs to improve the efficacy of a treatment, but the specific mechanisms by which the treatment is more effective must be addressed. This involves, for example, the investigation of possible synergistic effects between the material, microorganism, therapeutic delivered, and host tissues. To do this, *in vitro* and *ex vivo* assays might be useful. The development of more sophisticated human tissue models such as organoid-based or organ-on-chip technologies will be an invaluable source of information. In order to interface LTMs with these sophisticated models, more research needs to be focused on coculture methods that are stable for longer than 72 h. Drug dosage and therapeutic windows are other issues related to the lack of MoA knowledge. The practical doses of the drug reaching the target site are currently not accurately quantified. An overdose or repeated administration of these drugs could lead to toxic or unwanted side effects.

Third, manufacturing and quality control are critical parameters that must be considered. LTMs are usually customized to the patient needs, unlike traditional drugs produced in an industrial setting. Scaling-up production while maintaining viability (stability, storage), potency (biological activity), and identity (purity) of the microorganism is an important challenge that needs to be addressed. Therefore, batch-to-batch variations should be investigated.

Lastly, the diversity of LTMs might be hindering their translation to the clinic. LTMs are very varied in the sense that they are developed using different microorganisms, different genetic circuits, different polymers, and different manufacturing techniques. While this is positive and makes LTMs a great tool that could tackle many problems, it also makes the technology lacking in standardization. Results will differ from application to application, from strain to strain, from polymer to polymer, and so on. Therefore, standardization is more difficult than that for traditional drugs.

In conclusion, preclinical assessment of LTMs is growing and showing promising results, but there are still many critical parameters that need to be addressed concerning biocompatibility, safety, and efficacy to move this technology forward into the clinic. More efforts will need to be put into the standardization of LTM fabrication and high-throughput techniques that can assess LTMs in different scenarios to meet clinical requirements.
